# Breaking Down the Gut: A Case of Severe Toxin-Mediated Colitis

**DOI:** 10.7759/cureus.37092

**Published:** 2023-04-04

**Authors:** Duha M Al-Smadi, Manar Y Shahwan, Mahmoud Y Madi

**Affiliations:** 1 Internal Medicine, University of Sharjah, Sharjah, ARE; 2 Pathology and Laboratory Medicine, Saint Louis University School of Medicine, St. Louis, USA; 3 Gastroenterology and Hepatology, Saint Louis University School of Medicine, St. Louis, USA

**Keywords:** foodborne illness, bloody diarrhea, e.coli, infectious colitis, colitis

## Abstract

Shiga-toxin-producing Escherichia coli (STEC) is a worldwide, foodborne pathogen that can lead to life-threatening complications. Transmission has been associated with undercooked meat products, contaminated food and water sources, person-to-person contact, and direct exposure to infected farm animals. As the name suggests, the major virulence factors contributing to its pathogenicity are Shiga toxins, which can cause a spectrum of clinical presentations ranging from mild watery diarrhea to severe hemorrhagic colitis due to its toxic effects on the gastrointestinal tract. We report a case of a 21-year-old man seeking medical attention due to severe crampy abdominal pain and bloody diarrhea who was ultimately diagnosed with a less commonly encountered severe form of colitis in the setting of STEC infection. Thorough investigations while maintaining a high level of clinical suspicion allowed prompt medical care with a complete resolution of symptoms.

This case highlights the importance of having high clinical suspicion for STEC even with more severe forms of colitis and sheds light on the role of medical personnel in managing such cases.

## Introduction

Shiga-toxin-producing Escherichia (E.) coli (STEC) is a well-known cause of foodborne diarrheal illness secondary to hemorrhagic colitis. In the United States, approximately 63,000 cases are diagnosed annually [1]. Hemorrhagic colitis is characterized by crampy abdominal pain, diarrhea that may progress to frank bloody diarrhea, and occasionally, low-grade fevers [2].

STEC was first isolated in 1983 when two cases were reported around the same time. The first was a patient who consumed undercooked hamburgers and presented with bloody diarrhea. The second was a child with the hemolytic uremic syndrome (HUS). Escherichia coli (E.coli) O157:H7, the most common STEC, was later identified on the stool culture of both patients [3].

Managing E.coli-induced colitis begins with prompt and accurate laboratory testing. Stool samples are cultured on sorbitol MacConkey agar, which differentiates between pathogenic and non-pathogenic E.coli strains. A polymerase chain reaction is utilized to confirm the diagnosis by testing for antigens or toxins in the stool [1]. Treatment is centered around providing optimal supportive care and hydration for the patient, focusing on lowering the risk of possible complications [1]. It’s worth mentioning that people of all ages can get infected, but children younger than 10 years of age and older adults are particularly susceptible to severe infection leading to life-threatening complications like HUS and thrombotic thrombocytopenic purpura (TTP) [4].

Here, we present a case of a 21-year-old man who presented with abdominal pain and diarrhea, was found to have evidence of severe colitis, and was ultimately diagnosed with a severe form of STEC infection.

## Case presentation

A 21-year-old man with a past medical history significant for migraines, asthma, thalassemia minor, and inguinal hernia repair presented to the hospital with four days of abdominal pain, initially in the left lower quadrant of his abdomen, followed by diffuse pain most pronounced at the right upper quadrant and periumbilical area. The pain was stabbing, progressive in nature, given a score of nine out of 10 in severity, and was exacerbated by oral intake and attempted movement. The patient reported minimal relief in response to acetaminophen and ibuprofen. The pain was associated with nausea, decreased appetite, and frequent bloody loose stools up to 15 times daily. The patient denied vomiting, fevers or chills, melena, and any other symptoms on further review of systems. The patient denied prior similar symptoms in the past and denied travel outside the United States. On further questioning, the patient reported having consumed a beef sandwich from a street cart a few days prior to the onset of his symptoms. The patient was not chronically taking any medications and denied anticoagulant and non-steroidal anti-inflammatory drug use.

The patient reported a prior history of an esophagogastroduodenoscopy done at the age of 12 for nausea, which was reportedly normal. His family history was significant for colorectal cancer in his maternal grandmother and paternal grandfather. He denied a family history of inflammatory bowel disease or other autoimmune illnesses. The patient denied tobacco use and reported drinking two alcoholic beverages a week. He denied recreational drug use.

In the emergency department, the patient was afebrile with normal vital signs. On physical examination, there was moderate diffuse abdominal tenderness to palpation with a positive Murphy’s sign. No guarding, rebound tenderness, or rigidity was noted on examination. A digital rectal exam revealed bright red blood in the rectal vault with no visible external hemorrhoids or anal fissures.

Initial blood testing is summarized in Table 1. A stool culture and fecal calprotectin were ordered by the emergency medical team. A computed tomography scan of the abdomen and pelvis was obtained showing normal small and large bowels and evidence of gallbladder distention with mild pericholecystic fluid and inflammation raising suspicion for acute cholecystitis. This warranted further evaluation with an abdominal ultrasound, which ruled out this diagnosis. The patient was started on intravenous fluids, pain medications, and an antibiotic regimen with ceftriaxone and metronidazole.

**Table 1:** Laboratory values on initial presentation

Laboratory value	Reference range	Initial value
White blood cell count	4-11 K/uL	12.7 K/uL
Hemoglobin	13.5-17.5 g/dL	11.8 g/dL
Platelet count	150-400 K/uL	237 K/uL
Creatinine	0.7-1.3 mg/dL	0.86 mg/dL
Alkaline phosphatase	20-120 IU/L	56 U/L
Aspartate transaminase	0-45 IU/L	30 U/L
Alanine transaminase	0-45 IU/L	36 U/L
Total bilirubin	0-1.5 mg/dL	1.4 mg/dL
Fecal lactoferrin	Negative	Positive
Fecal calprotectin	10-50 μg/mg	319 μg/mg

On the following day, the patient’s pain progressed in severity with an increased frequency of bright red blood per rectum. Consultation with the gastroenterology service was completed with a decision to perform a colonoscopy to further evaluate the patient’s presentation. The patient's lack of improvement despite greater than three days of supportive measures, the progressive nature of his abdominal pain and bloody diarrhea despite high doses of oral and intravenous narcotic medications, and the patient's characteristics, including his age group and immune-competent state, raised concerns for diagnoses other than infectious colitis such as ischemic colitis and ulcerative colitis.

**Figure 1:**
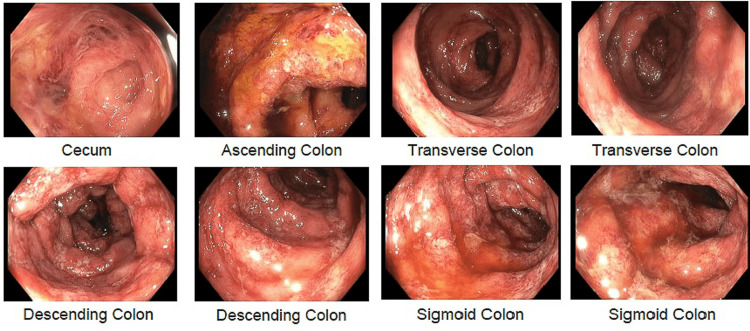
Colonoscopy findings Colonoscopy images revealed diffuse areas of severely erythematous, hemorrhagic, inflamed, and ulcerated mucosa consistent with severe colitis in the entire colon.

**Figure 2:**
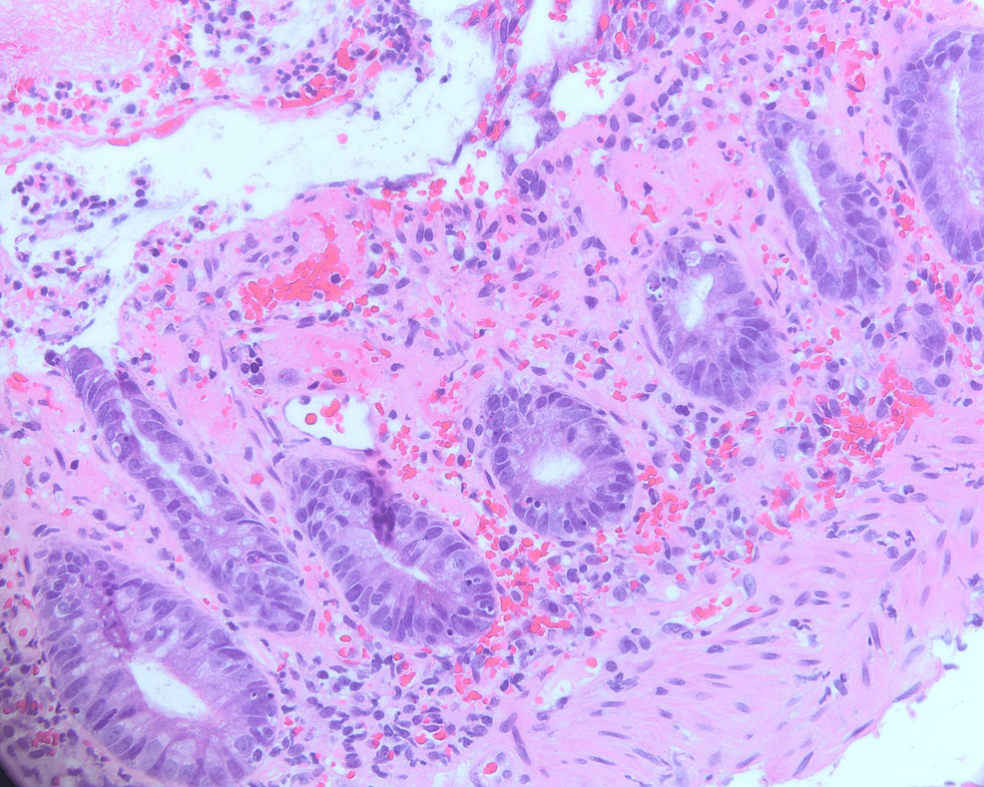
Pathology findings Moderate to severe mucosal injury characterized by crypt atrophy and lamina propria necrosis, increased apoptotic bodies, and acute inflammation associated with erosions and necrosis suggesting the diagnosis of acute colitis.

Colonoscopy revealed normal terminal ileum, diffuse areas of severely erythematous, hemorrhagic, inflamed, and ulcerated mucosa consistent with severe colitis in the entire colon with severe involvement of the left colon compared to the right with rectal sparing (Figure 1). Biopsies were taken with cold forceps for further histological evaluation. A pathological review of the biopsy specimens revealed moderate to severe mucosal injury characterized by crypt atrophy and lamina propria necrosis, increased apoptotic bodies, and acute inflammation associated with erosions and necrosis suggesting the diagnosis of acute colitis (Figure 2). At this point, stool culture showed positivity for E.coli Shiga-like toxin, confirming the diagnosis of acute colitis due to E.coli infection. The patient was treated with supportive measures and all antibiotics were discontinued.

On follow-up 48 hours later, the patient was doing well, tolerating oral fluids and a regular diet with a resolution of his symptoms, and was ultimately discharged home in excellent condition.

## Discussion

Escherichia coli, a gram-negative, cylindrical-shaped, flagellated, facultative anaerobic bacteria belonging to the Enterobacteriaceae family is a normal resident of the humans’ gastrointestinal tract that plays a crucial role in immunity and gut health [5]. Only when some strains acquire new genetic material do they become pathological and may cause serious gastrointestinal, urinary, and nervous system diseases [6]. Depending on their pathological scheme, E.coli is commonly classified into six different groups. These include enterotoxigenic E.coli associated with traveler’s diarrhea, enterohemorrhagic E.coli leading cause of hemorrhagic colitis and HUS, enteroaggregative E.coli causing persistent diarrhea, enteropathogenic E.coli causing infantile diarrhea, and enteroinvasive E.coli causing dysentery [6].

In this study, we focus on Shiga-toxin-producing E.coli also variably termed enterohemorrhagic E.coli due to their ability to cause hemorrhagic diarrhea.

Shiga toxin has two subunits: A and B. A subunit is enzymatically active and functions in inhibiting protein synthesis while the B subunit helps in the attachment to eukaryotic cells by binding to globotriaosylceramide on their surface. Shiga toxin is classified into Shiga toxin 1, which is almost identical to Shiga toxin produced by Shigella dysenteriae, and Shiga toxin 2, which has 57% homology to Shiga toxin 1. Shiga toxin 2 is the more virulent of the two and is more frequently associated with severe disease [7].

STEC infections are clinically categorized into either Shiga toxin 1 or Shiga toxin 2 encoding bacteria, with the latter being more virulent due to their association with bloody diarrhea and HUS [7].

E.coli O157:H7 is the most common STEC serotype studied. It became a major cause of foodborne illness leading to life-threatening complications such as HUS and TTP [1]. Unlike commensal E.coli, E.coli O157:H7 can withstand an array of harsh conditions due to its robust survival characteristics [1]. Studies of E.coli genetic material have revealed multiple virulence loci other than the Shiga toxin, contributing to its pathogenicities such as intimin, an attaching and effacement protein, hemolysin, a pore-forming toxin, and vesicles formed on the bacterial outer membrane, which facilitate the delivery of virulence factors and toxins to host cells [8-11]. However, the infectious dose of STEC can be as few as 10 viable bacteria, making the Shiga toxin the primary source of its virulence [1].

Epidemiological studies tracing back outbreaks of bloody diarrhea to domesticated animals revealed cattle to be the main reservoir of E.coli O157:H7. The transmission to humans occurs after the consumption of undercooked meat or unpasteurized dairy products. Human-to-human transmission is also possible through fecal shedding, accounting for 11% of infections. Contaminated water sources, inadequately washed vegetables and fruit, and direct contact with contaminated animals are among other ways of transmission as well [1]. Thereby, exploring possible exposures via thorough patient questioning can aid in properly diagnosing the patient.

STEC has become a major cause of foodborne illness worldwide not only due to the number of reported cases but also due to the severity of the illness and the associated deadly complications. In 2020, the Centers for Disease and Prevention in the United States reported a STEC incidence rate of 3.63 per 100,000 persons. Which dropped from 6.26 per 100,000 persons in 2019. This decrement in incidence rate might be explained by the safety measures applied due to the infamous severe acute respiratory syndrome coronavirus 2 pandemic [12].

Patients infected with STEC typically seek medical attention complaining of bloody diarrhea and crampy abdominal pain with a history of exposure to STEC through undercooked food or dairy products or after direct contact with farm animals. Early in the disease course, watery diarrhea is more common [1]. On physical examination, abdominal tenderness is reported and is usually more severe compared to other causes of enteritis, reflecting the intestinal inflammation and vasculitis caused by the Shiga toxin [1]. In the case presented above, exposure to undercooked beef was confirmed, which raised clinical suspension for foodborne illness and directed the medical care provided for the patient.

Patients presenting with STEC infection must be evaluated with high clinical suspicion, as prompt and accurate lab testing is critical for appropriate management. Initial evaluation with complete blood counts should be considered as the majority of E.coli O157:H7 colitis cases present with leukocytosis [1]. A stool sample should be obtained within the first days of the onset of symptoms. Furthermore, Shiga toxin can be detected in bloody diarrheal samples using enzyme-linked immunosorbent assays [1]. As the clinical presentation of STEC infection is non-specific and other differentials like inflammatory bowel disease, pseudomembranous colitis, ischemic colitis, and intussusception cannot be excluded initially, some cases might warrant further evaluation by means of colonoscopy with biopsies [11]. In our case, due to the age of the patient and the progressive and severe nature of the initial presentation, a colonoscopy was performed, and biopsies were taken for histological evaluation revealing severely inflamed and hemorrhagic colon consistent with severe colitis. This pathological pattern of focal superficial mucosal necrosis with acute inflammation and hemorrhage is like that of ischemic colitis.

The approach to managing STEC-induced colitis focuses on providing supportive care and maintaining hydration as most patients will recover within ten days without specific treatment other than intravenous fluids [1]. Treatment should also focus on lowering the risk of complications, especially HUS. Studies have proved that using antibiotic therapy in such cases can increase the risk of HUS, perhaps due to the rapid cell lysis and sudden release of Shiga toxins into the systemic circulation [1]. Given these findings, antibiotic therapy should generally be avoided.

## Conclusions

STEC infection should be considered in all patients presenting with crampy abdominal pain and bloody diarrhea with or without fever. History of possible exposure should be explored, as it can facilitate timely diagnosis. Laboratory testing should include complete blood counts and stool culture using MacConkey agar for detecting the presence of Shiga toxin or antigen in stool samples. Colonoscopy can be performed in severe cases or when other etiologies cannot be excluded as in the case presented here. Management is primarily supportive, as most patients will recover within 10 days without further interventions.
